# A generalization and an application of the arithmetic–geometric mean inequality for the Frobenius norm

**DOI:** 10.1186/s13660-018-1732-9

**Published:** 2018-06-20

**Authors:** Xuesha Wu

**Affiliations:** School of Marxism and General Education, Chongqing College of Electronic Engineering, Chongqing, P.R. China

**Keywords:** 15A42, 47A63, Unitarily invariant norms, Arithmetic–geometric mean inequality, Positive semidefinite matrices

## Abstract

Recently, Kittaneh and Manasrah (J. Math. Anal. Appl. 361:262–269, 2010) showed a refinement of the arithmetic–geometric mean inequality for the Frobenius norm. In this paper, we shall present a generalization of Kittaneh and Manasrah’s result. Meanwhile, we will also give an application of Kittaneh and Manasrah’s result. That is, we obtain an improvement of Jocić and Kittaneh’s inequality which was presented in (Jocić and Kittaneh in J. Oper. Theory 31:3–10, 1994).

## Introduction

Let $M_{n} ( C )$ be the space of $n\times n$ complex matrices. Let $\Vert \cdot \Vert $ denote any unitarily invariant norm on $M_{n} ( C )$. For $A \in M_{n} ( C ) $, the Frobenius norm of *A* is defined by $\Vert A \Vert _{F} = \sqrt {\operatorname{tr} ( {A^{*} A} )}$, where $\operatorname{tr} ( X )$ is the trace of *X*. It is known that the Frobenius norm is unitarily invariant.

Let $A,B \in M_{n} ( C )$ be positive semidefinite. Bhatia and Kittaneh proved in [1] that

1.1 $$ \bigl\Vert {A^{1/2} B^{1/2} } \bigr\Vert \le \biggl\Vert {\frac{{A + B}}{2}} \biggr\Vert , $$

which is known as the arithmetic–geometric mean inequality for unitarily invariant norms.

Let $A,X,B\in M_{n} ( C ) $ and suppose that *A* and *B* are positive semidefinite. Bhatia and Davis proved in [2] that

1.2 $$ \bigl\Vert {A^{1/2} XB^{1/2} } \bigr\Vert \le \biggl\Vert {\frac{{AX + XB}}{2}} \biggr\Vert , $$

which is a generalization of inequality (1.1).

Recently, Kittaneh and Manasrah [3] showed a refinement of inequality (1.2) for the Frobenius norm, which can be stated as follows:

1.3 $$ \bigl\Vert {A^{1/2} XB^{1/2} } \bigr\Vert _{F} + \frac{1}{2} \bigl( {\sqrt{ \Vert {AX} \Vert _{F} } - \sqrt{ \Vert {XB} \Vert _{F} } } \bigr)^{2} \le \biggl\Vert {\frac{{AX + XB}}{2}} \biggr\Vert _{F} . $$

The authors of [4] and [5] gave some generalizations of inequality (1.3).

Let $A,X,B\in M_{n} ( C )$ such that *A* and *B* are self-adjoint. Jocić and Kittaneh proved in [6] that for $n \in N$ and $j = 1, \ldots,n$,

1.4 $$ \bigl\Vert {A^{n + j} XB^{n - j + 1} - A^{n - j + 1} XB^{n + j} } \bigr\Vert \le \bigl\Vert {A^{n + j + 1} XB^{n - j} - A^{n - j} XB^{n + j + 1} } \bigr\Vert . $$

Bhatia gave a simple proof of inequality (1.4) in [7]. For more information on inequalities of unitarily invariant norms, the reader is referred to [8–12] and the references therein.

In this short note, we first present a new generalization of inequality (1.3). After that, as an application of inequality (1.3), we show a refinement of inequality (1.4) for the Frobenius norm.

## Main results

In this section, we show the main results of this paper. To do this, we need the following lemmas.

### Lemma 2.1

([11])

*Let* $A,X,B\in M_{n} ( C )$. *If* $\alpha \in [ {0,1} ]$, *then*

2.1 $$ \bigl\Vert {A^{*} XB} \bigr\Vert ^{2} \le \bigl\Vert {\alpha AA^{*} X + ( {1 - \alpha} )XBB^{*} } \bigr\Vert \bigl\Vert { ( {1 - \alpha} )AA^{*} X + \alpha XBB^{*} } \bigr\Vert . $$

### Lemma 2.2

*Let* $A,X,B\in M_{n} ( C )$. *Then*

$$\bigl\Vert {A^{*} XB} \bigr\Vert _{F} + \frac{1}{2} \bigl( { \sqrt{ \bigl\Vert {AA^{*} X} \bigr\Vert _{F} } - \sqrt{ \bigl\Vert {XBB^{*} } \bigr\Vert _{F} } } \bigr)^{2} \le \biggl\Vert {\frac{{AA^{*} X + XBB^{*} }}{2}} \biggr\Vert _{F}. $$

### Proof

By the polar decomposition of matrices and the properties of unitary invariant norms, we know that inequality (1.3) is equivalent to Lemma 2.2. This completes the proof. □

### Theorem 2.1

*Let* $A,X,B\in M_{n} ( C )$, $\alpha \in [ {0,1} ]$ *such that* *A*, *B* *are positive semidefinite*. *Then*

2.2 $$ \bigl\Vert {A^{1/2} XB^{1/2} } \bigr\Vert _{F} + \frac{1}{2} \bigl( {\sqrt {C ( \alpha )} - \sqrt{C ( {1 - \alpha} )} } \bigr)^{2} \le \biggl\Vert {\frac{{AX + XB}}{2}} \biggr\Vert _{F} , $$

*where*

$$C ( \alpha ) = \bigl\Vert {\alpha AX + ( {1 - \alpha} )XB} \bigr\Vert _{F} ,\qquad C ( {1 - \alpha} ) = \bigl\Vert { ( {1 - \alpha} )AX + \alpha XB} \bigr\Vert _{F}. $$

### Proof

By definition of the Frobenius norm, we have

$$\begin{aligned}& \begin{aligned} C^{2} ( \alpha ) &= \bigl\Vert {\alpha AX + ( {1 - \alpha } )XB} \bigr\Vert _{F}^{2} \\ &= \alpha^{2} \Vert {AX} \Vert _{F}^{2} + ( {1 - \alpha} )^{2} \Vert {XB} \Vert _{F}^{2} + 2\alpha ( {1 - \alpha} ) \bigl\Vert {A^{1/2} XB^{1/2} } \bigr\Vert _{F}^{2}, \end{aligned} \\& \begin{aligned} C^{2} ( {1 - \alpha} ) &= \bigl\Vert { ( {1 - \alpha} )AX + \alpha XB} \bigr\Vert _{F}^{2} \\ &= ( {1 - \alpha} )^{2} \Vert {AX} \Vert _{F}^{2} + \alpha ^{2} \Vert {XB} \Vert _{F}^{2} + 2 \alpha ( {1 - \alpha} ) \bigl\Vert {A^{1/2} XB^{1/2} } \bigr\Vert _{F}^{2}, \end{aligned} \end{aligned}$$

and so

2.3 $$ \Vert {AX} \Vert _{F}^{2} + \Vert {XB} \Vert _{F}^{2} - C^{2} ( \alpha ) - C^{2} ( {1 - \alpha} ) = 2\alpha ( {1 - \alpha} ) \Vert {AX - XB} \Vert _{F}^{2} . $$

It follows from (2.1) and (2.3) that

$$\begin{aligned}& \Vert {AX + XB} \Vert _{F}^{2} - \bigl( { \bigl( { \sqrt{C ( \alpha )} - \sqrt{C ( {1 - \alpha} )} } \bigr)^{2} + 2 \bigl\Vert {A^{1/2} XB^{1/2} } \bigr\Vert _{F} } \bigr)^{2} \\& \qquad {} - 2\alpha ( {1 - \alpha} ) \Vert {AX - XB} \Vert _{F}^{2} \\& \quad = \Vert {AX} \Vert _{F}^{2} + \Vert {XB} \Vert _{F}^{2} + 2 \bigl\Vert {A^{1/2} XB^{1/2} } \bigr\Vert _{F}^{2} - 4 \bigl\Vert {A^{1/2} XB^{1/2} } \bigr\Vert _{F}^{2} \\& \qquad {} - 2\alpha ( {1 - \alpha} ) \Vert {AX - XB} \Vert _{F}^{2}- \bigl( {\sqrt{C ( \alpha )} - \sqrt{C ( {1 - \alpha} )} } \bigr)^{4} \\& \qquad {} - 4 \bigl\Vert {A^{1/2} XB^{1/2} } \bigr\Vert _{F} \bigl( {\sqrt{C ( \alpha )} - \sqrt{C ( {1 - \alpha} )} } \bigr)^{2} \\& \quad = \Vert {AX} \Vert _{F}^{2} + \Vert {XB} \Vert _{F}^{2}- 2 \bigl\Vert {A^{1/2} XB^{1/2} } \bigr\Vert _{F}^{2} \\& \qquad {} - 2\alpha ( {1 - \alpha} ) \Vert {AX - XB} \Vert _{F}^{2}- \bigl( {\sqrt{C ( \alpha )} - \sqrt{C ( {1 - \alpha} )} } \bigr)^{4} \\& \qquad {} - 4 \bigl\Vert {A^{1/2} XB^{1/2} } \bigr\Vert _{F} \bigl( {\sqrt{C ( \alpha )} - \sqrt{C ( {1 - \alpha} )} } \bigr)^{2} \\& \quad \ge \Vert {AX} \Vert _{F}^{2} + \Vert {XB} \Vert _{F}^{2} - 2C ( \alpha )C ( {1 - \alpha} ) - \bigl( { \sqrt{C ( \alpha )} - \sqrt{C ( {1 - \alpha} )} } \bigr)^{4} \\& \qquad {} - 4 \bigl\Vert {A^{1/2} XB^{1/2} } \bigr\Vert _{F} \bigl( {\sqrt{C ( \alpha )} - \sqrt{C ( {1 - \alpha} )} } \bigr)^{2}-2\alpha ( {1 - \alpha} ) \Vert {AX - XB} \Vert _{F}^{2} \\& \quad = C^{2} ( \alpha ) + C^{2} ( {1 - \alpha} ) - 2C ( \alpha )C ( {1 - \alpha} ) - \bigl( {\sqrt {C ( \alpha )} - \sqrt{C ( {1 - \alpha} )} } \bigr)^{4} \\& \qquad {} - 4 \bigl\Vert {A^{1/2} XB^{1/2} } \bigr\Vert _{F} \bigl( {\sqrt{C ( \alpha )} - \sqrt{C ( {1 - \alpha} )} } \bigr)^{2} \\& \quad = \bigl( {C ( \alpha ) - C ( {1 - \alpha} )} \bigr)^{2} - \bigl( {\sqrt{C ( \alpha )} - \sqrt{C ( {1 - \alpha} )} } \bigr)^{4} \\& \qquad {} - 4 \bigl\Vert {A^{1/2} XB^{1/2} } \bigr\Vert _{F} \bigl( {\sqrt{C ( \alpha )} - \sqrt{C ( {1 - \alpha} )} } \bigr)^{2} \\& \quad = \bigl( {\sqrt{C ( \alpha )} - \sqrt{C ( {1 - \alpha} )} } \bigr)^{2} \bigl( {\sqrt{C ( \alpha )} + \sqrt{C ( {1 - \alpha} )} } \bigr)^{2} \\& \qquad {} - \bigl( {\sqrt{C ( \alpha )} - \sqrt{C ( {1 - \alpha} )} } \bigr)^{4}- 2 \bigl\Vert {A^{1/2} XB^{1/2} } \bigr\Vert _{F} \bigl( {\sqrt{C ( \alpha )} - \sqrt{C ( {1 - \alpha} )} } \bigr)^{2} \\& \quad = 4 \bigl( {\sqrt{C ( \alpha )} - \sqrt{C ( {1 - \alpha} )} } \bigr)^{2} \bigl( {\sqrt{C ( \alpha )C ( {1 - \alpha} )} - \bigl\Vert {A^{1/2} XB^{1/2} } \bigr\Vert _{F} } \bigr) \\& \quad \ge 0. \end{aligned}$$

That is,

$$\begin{aligned}& \bigl( {2 \bigl\Vert {A^{1/2} XB^{1/2} } \bigr\Vert _{F} + \bigl( {\sqrt{C ( \alpha )} - \sqrt{C ( {1 - \alpha} )} } \bigr)^{2} } \bigr)^{2} + 2\alpha ( {1 - \alpha} ) \Vert {AX - XB} \Vert _{F}^{2} \\& \quad \le \Vert {AX + XB} \Vert _{F}^{2}, \end{aligned}$$

which implies inequality (2.2). This completes the proof. □

### Remark 2.1

Putting $\alpha = 0$ or $\alpha = 1$ in inequality (2.2), we can obtain inequality (1.3). So, inequality (2.2) is a generalization of inequality (1.3).

Next, we will show a refinement of inequality (1.4).

### Theorem 2.2

*Let* $A,X,B\in M_{n} ( C )$ *such that* *A* *and* *B* *are self*-*adjoint*. *If* $n \in N$ *and* $j = 1, \ldots,n$, *then we have*

$$\bigl\Vert {A^{n + j} XB^{n - j + 1} - A^{n - j + 1} XB^{n + j} } \bigr\Vert _{F} +K^{2} ( {n,j} ) \le \bigl\Vert {A^{n + j + 1} XB^{n - j} - A^{n - j} XB^{n + j + 1} } \bigr\Vert _{F} , $$

*where*

$$\begin{aligned} K ( {n,j} ) =& \sqrt{ \bigl\Vert {A^{2} \bigl( {A^{n + j - 1} XB^{n - j} - A^{n - j} XB^{n + j - 1} } \bigr)} \bigr\Vert _{F} } \\ &{}- \sqrt{ \bigl\Vert { \bigl( {A^{n + j - 1} XB^{n - j} - A^{n - j} XB^{n + j - 1} } \bigr)B^{2} } \bigr\Vert _{F} }. \end{aligned}$$

### Proof

We prove it by induction. For $j=1$ and any positive integer *n*, by Lemma 2.2 and the triangle inequality for unitary invariant norms, we have

$$\begin{aligned}& \bigl\Vert {A^{n + 1} XB^{n} - A^{n} XB^{n + 1} } \bigr\Vert _{F} + \frac{1}{2}K^{2} ( {n,1} ) \\& \quad = \bigl\Vert {A \bigl( {A^{n} XB^{n - 1} - A^{n - 1} XB^{n} } \bigr)B} \bigr\Vert _{F}+ \frac{1}{2}K^{2} ( {n,1} ) \\& \quad \le \frac{1}{2} \bigl\Vert {A^{2} \bigl( {A^{n} XB^{n - 1} - A^{n - 1} XB^{n} } \bigr) + \bigl( {A^{n} XB^{n - 1} - A^{n - 1} XB^{n} } \bigr)B^{2} } \bigr\Vert _{F} \\& \quad = \frac{1}{2} \bigl\Vert {A^{n + 2} XB^{n - 1} - A^{n - 1} XB^{n + 2} + A^{n} XB^{n + 1} - A^{n + 1} XB^{n} } \bigr\Vert _{F} \\& \quad \le \frac{1}{2} \bigl\Vert {A^{n + 2} XB^{n - 1} - A^{n - 1} XB^{n + 2} } \bigr\Vert _{F} + \frac{1}{2} \bigl\Vert {A^{n + 1} XB^{n} - A^{n} XB^{n + 1} } \bigr\Vert _{F}, \end{aligned}$$

which is equivalent to

$$\bigl\Vert {A^{n + 1} XB^{n} - A^{n} XB^{n + 1} } \bigr\Vert _{F} + K^{2} ( {n,1} ) \le \bigl\Vert {A^{n + 2} XB^{n - 1} - A^{n - 1} XB^{n + 2} } \bigr\Vert _{F}. $$

Now, suppose that Theorem 2.2 has been proved for $j-1$. By Lemma 2.2, the triangle inequality for unitary invariant norms, and induction hypothesis, we have

$$\begin{aligned}& \bigl\Vert {A^{n + j} XB^{n - j + 1} - A^{n - j + 1} XB^{n + j} } \bigr\Vert _{F} + \frac{1}{2}K^{2} ( {n,j} ) \\& \quad = \bigl\Vert {A \bigl( {A^{n + j - 1} XB^{n - j} - A^{n - j} XB^{n + j - 1} } \bigr)B} \bigr\Vert _{F} + \frac{1}{2}K^{2} ( {n,j} ) \\& \quad \le \frac{1}{2} \bigl\Vert {A^{2} \bigl( {A^{n + j - 1} XB^{n - j} - A^{n - j} XB^{n + j - 1} } \bigr) + \bigl( {A^{n + j - 1} XB^{n - j} - A^{n - j} XB^{n + j - 1} } \bigr)B^{2} } \bigr\Vert _{F} \\& \quad = \frac{1}{2} \bigl\Vert {A^{n + j + 1} XB^{n - j} - A^{n - j} XB^{n + j + 1} + A^{n + j - 1} XB^{n - ( {j - 1} ) + 1} - A^{n - ( {j - 1} ) + 1} XB^{n + j - 1} } \bigr\Vert _{F} \\& \quad \le \frac{1}{2} \bigl\Vert {A^{n + j + 1} XB^{n - j} - A^{n - j} XB^{n + j + 1} } \bigr\Vert _{F} + \frac{1}{2} \bigl\Vert {A^{n + j - 1} XB^{n - ( {j - 1} ) + 1} - A^{n - ( {j - 1} ) + 1} XB^{n + j - 1} } \bigr\Vert _{F} \\& \quad \le \frac{1}{2} \bigl\Vert {A^{n + j + 1} XB^{n - j} - A^{n - j} XB^{n + j + 1} } \bigr\Vert _{F} + \frac{1}{2} \bigl\Vert {A^{n + j} XB^{n - j + 1} - A^{n - j + 1} XB^{n + j} } \bigr\Vert _{F}, \end{aligned}$$

which is equivalent to

$$\bigl\Vert {A^{n + j} XB^{n - j + 1} - A^{n - j + 1} XB^{n + j} } \bigr\Vert _{F} +K^{2} ( {n,j} ) \le \bigl\Vert {A^{n + j + 1} XB^{n - j} - A^{n - j} XB^{n + j + 1} } \bigr\Vert _{F}. $$

This completes the proof. □
